# Models of Protocells Undergoing Asymmetrical Division

**DOI:** 10.3390/e26040281

**Published:** 2024-03-26

**Authors:** Marco Villani, Elena Alboresi, Roberto Serra

**Affiliations:** 1 Department of Physics, Informatics and Mathematics, Modena and Reggio Emilia University, 41121 Modena, Italy; elenaalboresi@gmail.com (E.A.); rserra@unimore.it (R.S.); 2 European Centre for Living Technology, 30123 Venice, Italy; 3 Institute of Advanced Studies, University of Amsterdam, 1012 WX Amsterdam, The Netherlands

**Keywords:** protocell, synchronization, asymmetric replication, populations, models, sustained reproduction

## Abstract

The conditions that allow for the sustained growth of a protocell population are investigated in the case of asymmetrical division. The results are compared to those of previous studies concerning models of symmetrical division, where synchronization (between duplication of the genetic material and fission of the lipid container) was found under a variety of different assumptions about the kinetic equations and about the place where molecular replication takes place. Such synchronization allows a sustained proliferation of the protocell population. In the asymmetrical case, there can be no true synchronization, since the time to duplication may depend upon the initial size, but we introduce a notion of homogeneous growth that actually allows for the sustained reproduction of a population of protocells. We first analyze Surface Reaction Models, defined in the text, and we show that in many cases they undergo homogeneous growth under the same kinetic laws that lead to synchronization in the symmetrical case. This is the case also for Internal Reaction Models (IRMs), which, however, require a deeper understanding of what homogeneous growth actually means, as discussed below.

## 1. Introduction

The process of cell division (fission) is extremely important both for unicellular species, where it provides the mechanism of proliferation, and for multicellular organisms, where it is active not only during embryo growth to full adult size but also during the whole lifetime of an individual, assuring proper renewal of its cells.

Cell fission is usually preceded by duplication of its genetic material, to ensure that every daughter cell gets a full copy (a remarkable exception being the generation of germ cells in those species that undergo sexual reproduction). While present-day cells host sophisticated control mechanisms that assure that fission does not start before DNA duplication has occurred [1], it is highly unlikely that such control mechanisms were in place in the early days of primordial protocells.

Protocells are entities that resemble, in some way, but are much simpler than, present-day cells Besides their possible role in the origin of life from abiotic material, the interest in protocells is related also to their possible application in different domains (e.g., drug synthesis and delivery, remediation of polluted sites, etc.). Many different hypotheses have been proposed concerning their “architectures” as well as their chemical compositions and the relevant kinds of physical and chemical processes that take place. A key property should of course be their capability to grow and reproduce, giving rise to daughter protocells that resemble their parents. It is fair to say that, while several interesting intermediate results have been obtained [2,3,4], full-fledged protocells, able to continuously generate several successive generations, have not yet been achieved. Given the time and cost of actual wet experiments, mathematical and computational models are extremely important to indicate directions of research and to test the suitability of the different proposals. These models are also important since they allow one to experiment freely with different parameter values, which can be difficult to achieve, and to observe the values of all the variables, including those that are difficult to measure in a laboratory experiment. While they cannot substitute laboratory experiments, they can be of great help in identifying the main strengths and weaknesses of the different proposals, and in pointing out major problems that need to be addressed [5,6,7].

There is an interesting class of supramolecular structures, i.e., lipid vesicles, which do actually exist, and which are spontaneously formed under a broad set of conditions, in aqueous solutions of amphiphiles [8,9,10,11,12]. Such lipid vesicles (sometimes called liposomes) resemble cells in that their aqueous interior is surrounded by an approximately spherical membrane, which is composed of a lipid bilayer, similar to those found in cells and in some cellular compartments. If further lipids are supplied, the size of vesicles can grow and, under some experimental conditions, their splitting has been observed [2,13,14,15,16]. The resemblance of this process to cell fission is probably the main reason why vesicles have been proposed as the starting point of most hypothesized protocell architectures.

In this paper, we will indeed consider broad classes of mathematical and computational models of protocells, all based upon lipid vesicles that will be assumed to spontaneously undergo fission when they reach a certain size. Moreover, it will be assumed that each protocell hosts some chemicals (“replicators”) that are able to collectively self-replicate, and that some of these replicators also increase the rate of growth of the vesicle lipid membrane (e.g., by catalyzing the synthesis of its amphiphiles - as it is often the case, we will often use the term lipid as a synonym of amphiphiles: lipids with a polar head are indeed the best-known examples of amphiphilic molecules). For simplicity, we consider a single type of lipid in aqueous environments, so the replicators determine the identity (i.e., the properties) of the protocell: their set (or a subset) can be loosely regarded as its “protogenetic material”.

Two different processes take place in these vesicles, i.e., (i) cell reproduction by fission and (ii) duplication of the genetic material. One major problem is indeed that of assuring that the two processes take place at the same pace. If cell reproduction were much faster than duplication, the genetic material would be increasingly diluted through generations, while in the opposite case, its quantity would continue to increase and accumulate in cells. In both cases, no sustainable growth of a population of protocells would take place.

If we do not want to introduce unlikely hypotheses about the existence from the very beginning of sophisticated control mechanisms, we are led to raise the key question of whether the synchronization of these two processes might have spontaneously emerged in early (proto)life. In our previous works, briefly recalled in Section 2, we used simplified mathematical and computational models (which will be collectively referred to as here as Symmetrical Division Models) to show that this may indeed be the case under a surprisingly wide set of different hypotheses. The rates of the two processes (reproduction of the protocell and duplication of its protogenetic material) tend to a common value generation after generation, without resorting to any specific evolutionary mechanism, provided that they are coupled. We referred to this tendency as the synchronization of the two processes, and we showed that it is robust with respect to different types of random fluctuations.

The models that we have studied are fairly abstract, as they do not rely on specific hypotheses about the chemical nature of the replicators, and they can accommodate different types of kinetic equations. On the other hand, they are abstractions of more specific models, so that a property that holds for them will hold also for the specific models, provided that other simplifying assumptions (such as those of spherical protocells, of uniform concentrations, or of a fixed threshold for fission) hold. These aspects are further discussed in Section 5.

In these models, the dynamics of a protocell between its birth and its fission is ruled by ordinary first-order differential equations, which allow us to compute the relationship between the initial quantities of replicators ***X*** at successive generations, i.e., the discrete map that relates ***X****(k + 1)* to ***X****(k)*. We make the simplifying assumption that cell division takes place when a fixed size has been reached. There are interesting models of this process, but here we simply assume that it is “fast” with respect to the growth of the protocells and that it gives rise to two spherical daughter protocells Various hypotheses can be made about the division process. For example, the sphere deforms, giving rise to an elongated shape that then gives rise to two spheres, without losing any lipid. Assuming that this process is “fast”, we have carried out simulations in which no internal material is lost, observing that even in this case, homogeneous growth is obtained. Since the size of the mother cell when splitting takes place is fixed, as well as the initial size of the newborns, the various generations differ only in the initial values of the replicators. Therefore, in order to prove synchronization, it suffices to prove that as *k* grows, the quantities of replicators (and therefore the lifetime of each generation) tend to develop toward constant values. In many interesting cases, this can indeed be analytically demonstrated; in other cases, this can be verified by numerical simulations. 

The situation seems reasonably well understood. In the case of symmetrical division, and here below we summarize in Section 2 the main results of these previous studies. However, different phenomena have also been observed in real vesicles, which may sometimes give rise to offspring of largely different sizes [17]. In this paper, we will consider the case where a protocell splits into two daughters of different sizes, using models similar to those that had been previously applied to the case of symmetrical division. We will consider both Surface Reaction Models (SRMs), where the replicators are found in the lipid membrane, and Internal Reaction Models (IRMs), where they inhabit the internal aqueous phase. These models are quite abstract (for example, replicators are defined by their kinetic equations, without any explicit reference to their chemical identity) so they can represent several different more specific models.

When the offspring are born differently, the issue can no longer be that of synchronization, since, in general, they will mature (i.e., reach the critical size for division) at different times. Sustainable growth through generations will take place if the daughters are similar to how their parents were when they were born. We will refer to this situation as *homogeneous growth*: synchronization implies homogeneous growth, but homogeneous growth can be achieved even without the synchronization of the two processes. In order to claim that a generation is similar to the previous one, it may be requested that, at splitting time, the chemical compositions of the cells be the same. Since we assume that there is a single type of lipids, and that the size of splitting is the same, the chemical composition of a cell is determined by the total quantities of different replicators. We therefore observe *homogeneous growth* if these quantities are the same at the end of successive generations. For reasons that will be detailed in Section 4, it will actually be required that the ratios of the quantities of different types of replicators be the same in different protocells.

We are of course aware that evolution requires that changes can intervene between generations. The kind of abstract models described here and in the previous works on synchronization are based on deterministic differential equations among a fixed set of replicators and lipids, so there is no explicit room for true evolution, which requires the introduction of further rules (e.g., those for the creation of new chemical species). We had also previously shown how this can be done, proving that synchronization can be observed in stochastic evolving models, in the case of symmetrical division [18,19,20,21]. We suppose that homogeneous growth will also be observed in the case of evolution under asymmetrical division, but these studies lie beyond the scope of this paper.

The purpose of this paper is indeed to study under which conditions homogeneous growth can take place when the daughter cells are of different sizes. In order to do so, we will consider the case of a population that repeatedly undergoes the same kind of asymmetrical division, looking for the possible emergence of a sustainable pattern of protocells. In the simpler case of symmetrical division with synchronization, the time interval between successive fissions tends to develop toward a common value, so the population size doubles at each interval, undergoing exponential growth. In asymmetrical division, the duplication time is a function of the initial quantities of lipids (*C*) and of replicators *(X*_1_*…X_q_)*: while the initial and final quantities of lipids are the same for every protocell that has the same size, the initial compositions of replicators may depend upon their previous histories, so different protocells can duplicate at different times. Each splitting event gives rise to a small protocell and to a large one, while of course the mother disappears; therefore, the total number of protocells increases by one. As the population size grows, the time interval between two successive splitting events tends to shrink, thus increasing the overall growth rate of the population.

We performed various types of simulations, starting in each case from a single protocell size of *θ*/2 and a given quantity of replicators. The above-mentioned unbounded growth of the population size is of course unrealistic, since the scarcity of some resource will sooner or later prevent any further growth. In order to analyze the long-time behavior of the system, we took the simple approach of imposing a limit on the total number of protocells: when a new splitting would lead to exceeding (by one) the limit, the two newborns are added to the existing population, and a randomly chosen protocell is removed—thus keeping their total number constant. We report here results based on this method, although we have also performed different types of simulations, e.g., following a single lineage through generations, which confirm the main conclusions. Note that the populations are renewed, but there is no selection pressure, since the removal is performed randomly, with uniform distribution. The approach taken here resembles the so-called Moran process [22], used to study the evolution of populations of fixed size, composed of two different types of individuals. Indeed, we use asynchronous update, and we keep the population size constant; we can also identify the two types of individuals that make up the population as those of protocells that are born large (type A) and small (type B). The fact that the lifetime of individuals of type A is shorter than that of type B may also be related to (some kind of) fitness. However, in our approach, the two different types are always generated together, so there is never true extinction.

In Section 3 we will introduce the model of asymmetrical division in SRMs, and we will first analyze the case of a single self-replicator, whose proliferation is described by a linear differential equation. We will also consider the case when more replicators interact linearly, so that self-replication is a collective rather than individual property. We will also analyze some nonlinear cases. It will be shown (perhaps unexpectedly) that in all these cases the behavior under asymmetrical division is strikingly similar to that of its symmetrical counterpart, in the sense that homogeneous growth is observed in those cases where synchronization is observed in symmetrical models. 

In Section 4 we then study asymmetrical division in models where reactions take place in the aqueous interior of the protocell, paying particular attention to the important case of two replicators catalyzing each other’s synthesis. It will be seen that in such IRMs, some differences are observed with respect to symmetrical division, and they will be stressed. 

The results will be discussed in Section 5, where indications for further work will also be presented.

## 2. Symmetrical Division Models

Most protocell models deal with symmetrical division, where the fission of a protocell gives rise to two daughter protocells of equal size (see e.g., [23,24,25]). We will quickly summarize here below the models that we had previously used to study synchronization under symmetrical division, which will be generalized in this paper to asymmetrical cases. No new results will be presented in this section. To the best of our knowledge, the importance of synchronization was first observed [26] in the case of the so-called Los Alamos bug model [27,28,29,30], which was based on a number of specific hypotheses about the protocell architectures and the chemical properties of the replicators. Shortly after, it was shown [31] that the same properties might hold in more general cases by introducing a new class of abstract models, amenable to analytical treatment or to numerical simulation. In a series of papers, it was shown that synchronization can be found in several cases, and that it is robust with respect to different changes [20,31,32,33,34]. These results have also been discussed in depth in a book [21].

As described in Section 1, in this work, protocells are assumed to be spherical, and to undergo duplication when they reach a critical size *θ*, giving rise to two equal daughter cells. There is a single type of lipids and, while there may be different types of replicators, for the sake of simplicity, we will first consider the case of a single (self)replicator. Let *C* be the total quantity of membrane lipids, and let *X* be the total quantity of replicator; then, a protocell grows in time according to two ordinary differential equations:

(1) $$\{\begin{array}{c} \dot{C}=f(C,X)\\ \dot{X}=g(C,X) \end{array}$$

where a dot denotes a time derivative. Let *S* be the surface area and let *δ* be its (constant) width; then, the volume of the lipid membrane is *V_m_* = *Sδ*. Since we assume spherical protocells, *S* also determines the total volume of the protocell, i.e., its size. Moreover, if lipid density *ρ* is constant, the total quantity of membrane lipid *C* is equal to *Sδρ*. Therefore, the critical size is associated with a specific value of *C*, so we assume that splitting takes place when *C* = *θ.* In Appendix A, you can find a table with all the symbols used in the paper, and their brief description.

The growth of both *X* and *C* depends also on the availability of suitable precursors, which is given for granted here (i.e., they are buffered). Other assumptions are also necessary to arrive at Equation (1); they are discussed at length in [31] and in [21] and will not be further analyzed here. From now on, in this section, we will concentrate on Surface Reaction Models, where the replicators are found in the membrane. In the following, time is measured in arbitrary units; the absolute quantities in kilograms; lengths in meters; and concentrations in kg/liter. The units of measurement of the other entities (for example, those of parameters) are consistently derived. Let us assume that *X* linearly catalyzes its own production and also the growth of the membrane from suitable precursors. In this case, Equation (1) becomes:

(2) $$\left\{\begin{array}{c} \dot{C}=\alpha X\\ \dot{X}=\eta X \end{array}\right.$$

Observing that *Q(t)* ≡ *ηC(t)* – *αX(t)* is constant during the continuous growth described by Equation (2), and observing that in each generation the initial and final sizes are *θ*/2 and *θ*, one straightforwardly derives a relationship between the initial values of the replicator quantity in successive generations:

(3) $$X_{k+1}=\frac{X_{k}+D}{2}\;;\;D=\frac{\theta \eta }{2\alpha }$$

By taking the limit *k* → *∞,* one gets *X_k_* → *D*; since the initial values of *X* become constant, so does also the duplication time Δ*T_k_*:

(4) $${\Delta T}_{k}\to \frac{1}{\eta }ln2$$

Synchronization is thus proven. Using a similar method, it can also be analytically proven if *dX*/*dt* follows a nonlinear power law, i.e., when the kinetic equations are the following:

(5) $$\left\{\begin{array}{c} \dot{C}=\alpha X\\ \dot{X}=\eta C^{\nu -1}X^{\nu } \end{array}\right.$$

provided that *ν* < 2.

As it has been observed, autocatalysis is quite rare; therefore, it is interesting to consider cases where more replicators are involved. Let $\vec{X}$ denote the quantities of the *q* types of replicators *X_1_ …X_q_* and let the kinetic equations be as follows:

(6) $$\left\{\begin{array}{c} \dot{C}=\vec{\alpha }\cdot \vec{X}\\ \dot{X}=M\vec{X} \end{array}\right.$$

The long-time behavior of the system is ruled by the eigenvalue with the largest real part (*λ*_1_) of the q·q matrix ***M***. If the matrix ***M*** is nonnegative (all its entries are ≥0) and non-null (at least one entry is ≠0), then the Perron theorem guarantees that *λ*_1_ is positive and admits a nonnegative eigenvector (whose components specify the long-term quantities of the various replicators). In this case, synchronization is guaranteed, a conclusion that also holds if there are some negative diagonal terms. The discussion of cases with negative nondiagonal terms requires more care, but a satisfactory physical interpretation can also be given, as extensively discussed in [18,21,32,33].

This is how far we can go with analytical methods. We can, however, also investigate different cases of one or several interacting replicators using simulations, and we find that synchronization is widespread. It is not achieved in the case of nonlinear quadratic interactions, but this result is structurally unstable: if a lower order term (e.g., linear) is added, then synchronization takes place even when there are quadratic terms. In some cases, *λ*_1_ is complex, and synchronization takes place between quantities that oscillate in time. We also tested some kinetic equations that are known to give rise to chaotic behavior, but when replicators are coupled to the splitting of their lipid containers, synchronization is again observed [34].

Apart from changes in the kinetic equations, synchronization has also been observed when the full geometry of the vesicle is taken into account, when the membrane itself is composed of replicators (as, for example, in GARD models [35,36]), and when the splitting threshold is subject to random fluctuations. Synchronization is robust with respect to these perturbations.

Stochasticity can play a major role when the number of specimens of a type of replicator is very small, as it may easily happen when a new type is discovered; in these cases, the deterministic kinetic equations used so far, based on the law of mass action, may be inadequate, and they should be substituted with stochastic equations. We have also analyzed this type of equation, showing that synchronization may also take place (e.g., when there is a so-called RAF set of replicators [20,37,38]) but a thorough discussion of these cases lies beyond the purpose of this paper.

The other major class of models that will be considered are those where the replicators are solutes in the internal liquid phase, which are also able to catalyze the growth of the container from buffered precursors (indeed, most protocell models assume that the key reactions take place inside the protocell, rather than in the membrane). 

Like in the SRM case, we will assume a homogeneous distribution of the replicators inside and outside of the protocell, which implies their infinitely fast diffusion rate in water. We will also assume that the external concentrations of precursors are unaffected by flows to and from the protocells, and that their transmembrane diffusion is infinitely fast, so that precursor concentrations are buffered. These simplifying assumptions can be relaxed, and they have been relaxed, in Symmetrical Division Models [32].

Assuming that both the growth of the container *C* and the rate of self-replication are linear functions of the concentration of *X*, the equations of the IRMs turn out to be the same as those for SRMs, i.e., Equation (5) (which is the same as Equation (2) in the case of a single replicator). The observed phenomena are therefore the same as those of the SRMs.

In the case of nonlinear models, the equations are no longer the same. Let us consider the case of first-order reactions, which involve a single type of reactant: according to the law of mass action, when precursors are always available, the reaction rate (equal to the rate of change of [*X*] *= X*/*V_i_*, where *V_i_* is the internal volume) is proportional to [*X*]. The total number of events in unit time is proportional to [*X*]*V_i_*, i.e., to the internal quantity *X*. This is why, in the linear case, the equations are the same as those of SRMs.

Note that in second-order reactions the rate of encounters per unit volume is proportional to the product of concentrations, and therefore to (*X*/*V_i_*)^2^, so that the total number of encounters in the whole protocell volume is proportional to *X*^2^/*V_i_*. Assuming that the internal volume is approximately proportional to *S*^3/2^, and therefore to *C*^3/2^, then the equations become the following:

(7) $$\left\{\begin{array}{c} \dot{C}=\alpha X\\ \dot{X}=\eta C^{\frac{3}{2}}X^{2} \end{array}\right.$$

Despite this difference, the results concerning synchronization still follow the same pattern as those for IRMs, as detailed in [21,32]. A remarkable difference with respect to SRMs is that, in this case, some internal material is lost (see the following Section 4 for details).

## 3. Asymmetrical Division in Surface Reaction Models

The notions of SRM and IRM concern the architecture of a protocell, and they can of course be applied also to the study of asymmetrical replication. The continuous growth phase of SRMs is described by the same equations as those of the previous section, while the splitting may give rise to two different daughters. As in the case of symmetrical division, we will assume that no lipids are lost, so the total volume of the membranes of the two daughters equals that of their “mother”. Since the width *δ* of the membrane is constant, this also implies that the total surface is constant, which in turn (assuming uniform concentration) implies that the total quantity *C* is conserved in splitting. Note that, since we assume that there is a single type of lipids, using different units (e.g., mass or moles or number of molecules) to measure this quantity does not make any conceptual difference, since they are all related to each other by constant multiplicative coefficients. Just like in the models of Section 2, *C = Sδρ* and the condition for splitting is *C = θ.*

In asymmetrical division, one daughter gets a fraction *ω* of the total membrane lipids, i.e., *ωθ* [39]. Replicators are homogeneously dissolved in these lipids; therefore, that protocell will also get the same fraction of replicators, i.e., *ωX_fin_* where *X_fin_* is the total quantity of X in the mother protocell at splitting time. The other daughter will then inherit (1 − *ω*)*θ* lipids and (1 − *ω*)*X_fin_* replicators.

In the case of symmetrical division, we had written equations using quantities of replicators in a protocell. But here, each division gives rise to a large and to a small protocell, and using concentrations, as is usually done in chemistry, turns out to be convenient. Note that the splitting threshold is fixed irrespective of the initial size; therefore, concentrations at splitting time are simply proportional to quantities.

We performed several simulations of protocell populations. For reasons highlighted in Section 1, all these populations start from the asymmetrical division of a mother protocell; the fraction ω is the same at every generation so the impact of its value can be determined (but see Section 5 for a preliminary discussion about the case of a variable threshold). There is a limit *N_max_* on the maximum number of protocells in the system, so after a transient their total number remains constant (as discussed in Section 1, existing protocells are removed randomly—with uniform distribution—to avoid exceeding that number). When that number has been reached, a generation is defined to be equal to *N_max_* individual splitting events (i.e., the minimum number that might allow for a complete renewal of the population).

At the moment of scission, the daughter protocells inherit a fixed fraction of lipids from the mother protocell (let them be *ωθ* and (1 *− ω*)*θ*), but the initial concentrations [*X_initial_*] of replicators may differ in different scission events, depending upon the different histories of the mother protocell. The lifetime of protocells (the duration of time between the birth of a protocell and its conclusion due to fission) might also be different since it also depends upon [*X_initial_*].

Transients may depend upon initial values, so we will show below results concerning the distribution of the relevant variables after some generations have elapsed since the division of the first protocell (and since reaching the maximum number of protocells *N_max_*).

**Figure 1:**
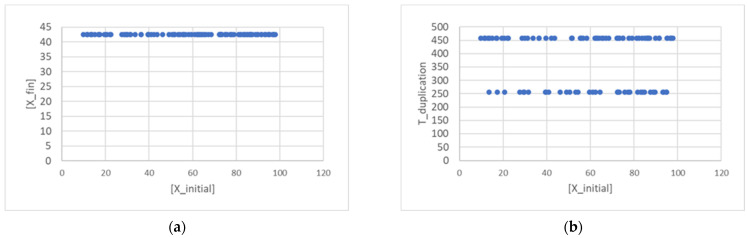
Final concentration of *X* (**a**) and duplication times (**b**) at the 75th generation (15,000 duplications in a population of 200 individuals, asymmetrical division, *ω* = 0.4). The X-axis reports the *X* concentration values of the initial generation. In this figure, *α* = 0.05, *η* = 0.002, *θ* = 2.70 × 10^−16^.

Let us first consider the case of a single linear replicator, whose continuous growth is described by Equation (2). The first interesting observation is that the final concentration tends to develop toward a constant value, the same for every protocell, which depends upon the values of the ratio *ηθ*/*α* like in the case of symmetrical division (Section 2). There are now two lifetimes, one for the protocells that were born small, and one for those that were born large. It is interesting that, after 75 generations, the values of the final concentration [*X_fin_*] and of the lifetime *T_duplication_* are the same, irrespective of the value of the concentration of the progenitor cell (provided of course that the other parameters are kept fixed), as shown in Figure 1. In Figure 1, as in all subsequent ones, we show the results of a particular choice of parameters. We ran simulations using a wide variety of parameter combinations, (in particular, varying the threshold *θ* several times, and the coupling with the container and the catalysis coefficients by orders of magnitude) always obtaining essentially the same trends.

One can observe that the concentration at the beginning of a new generation is the same as the concentration of the previous one just before splitting, so the initial concentration of one daughter, *X_initial_*/*C_initial_*, is as follows:

(8) $$\frac{X_{initial}}{C_{initial}}=\frac{\xi X_{fin,previous}}{\xi \theta }=\frac{X_{fin,previous}}{C_{fin}}$$

where *ξ* = *ω* for one daughter, *ξ* = (*1* − *ω*) for the other one, and *X_fin,previous_* refers to the previous generation. Since the final concentration becomes constant, so do the (equal) concentrations of the two daughters. In SRMs, after an initial transient, all the initial and final concentrations take the same value, and it has been observed in simulations that they also remain constant during the continuous growth phases. Indeed, concentrations tend to develop toward the value *X*/*C = η*/*α*, which makes their time derivatives vanish since

(9) $$\frac{d}{dt}\left(\frac{X}{C}\right)=\eta \left(\frac{X}{C}\right)-\alpha {\left(\frac{X}{C}\right)}^{2}$$

Also note that in the long term, the sum of final concentrations of replicators in the two daughters is equal to the same sum at the previous generation, thus allowing homogeneous growth. 

A major observation is that the case of a single linear replicator is one of homogeneous growth—it behaves in a way similar to that of symmetrical division, where there is synchronization. As it is obvious (also checked by simulation), the same also applies to the case of several linear replicators, where the eigenvalue with the largest real part and its eigenvector(s) determine the outcome. 

**Figure 2:**
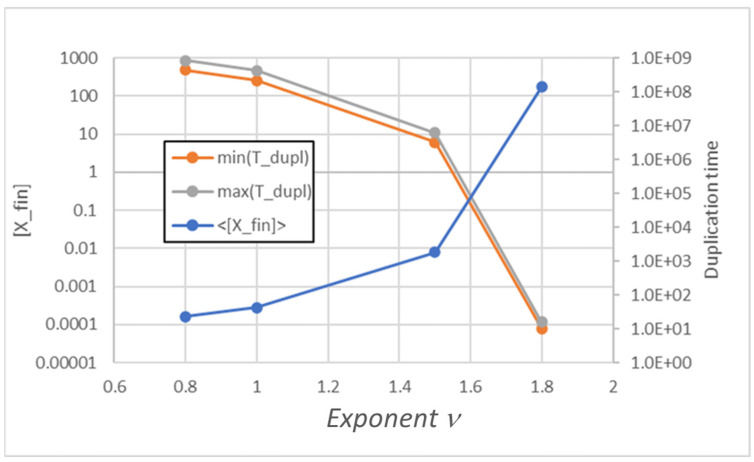
Duplication times *T_duplication_* and replicator concentration at duplication time [*X_fin_*] of a stable population, as *ν* varies.

Moreover, also in the simulated cases of nonlinear replicator kinetics, one observes a similar parallelism between the behaviors of the two types of models. Note that in the case of a power law, when *d*[*X*]/*dt* is proportional to [*X*]*^ν^*, we see homogeneous growth as long as *ν* < 2 (see Figure 2).

The overall conclusion of these studies is that asymmetrical SRMs behave much like Symmetrical Division Models: they lead to homogeneous growth in those cases where there was synchronization, thus allowing the growth of a population of protocells that, after a transient, maintain their features. Of course, mutations have not yet been considered in the studies described in this paper.

**Figure 3:**
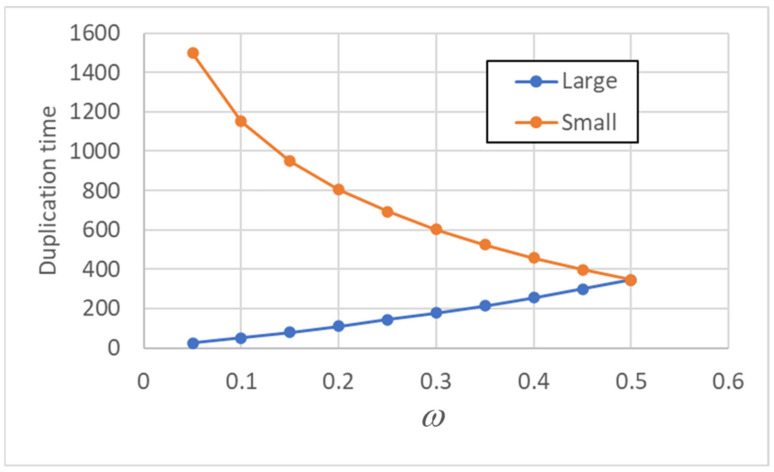
Duplication times *T_duplication_* (of a stable population—here is the 75th generation) as asymmetry varies: the duplication times of “born small” and “born large” protocells are shown. Values on the x-axis show the fraction of lipids inherited by the smaller protocell.

It is also interesting to explore how the variables depend upon the degree of asymmetry, related to the fraction *ω*. The larger protocells reach the splitting threshold sooner than the smaller ones, and in Figure 3, it is shown how the difference between the duplication times increases as asymmetry increases (linear case).

## 4. Asymmetrical Division in Internal Reaction Models

In this case, the replicators are dissolved in the internal water phase; as in the previous case, splitting is achieved by partitioning the membrane between the two daughters, without any loss of lipids. Since for simplicity we assume that both the mother’s and the daughters’ shapes are spherical, then, for every protocell, the volume *V* is related to the quantity of membrane lipids *C* by the following:

(10) $$V\left(C\right)=\frac{4}{3}\pi \delta^{3}{\left(\frac{\sqrt{\frac{C}{\rho \pi \delta^{3}}-\frac{1}{3}}-1}{2}\right)}^{3}\sim \frac{1}{6}\pi \delta^{3}{\left(\frac{1}{\rho \pi \delta^{3}}\right)}^{\frac{3}{2}}C^{\frac{3}{2}}=kC^{\frac{3}{2}}$$

where *k* is a constant. The last expression is valid when the membrane width *δ* is small, and we see that in this case, the Volume scales as C^3/2^. When the membrane reaches the value *C = θ* (so *V*(*C*) = *kθ*^3/2^), the cell is divided into two fractions, whose membranes are proportional to *ω* and to (1 − *ω*); therefore, the corresponding volumes are proportional to *ω*^3/2^ and to (1 − *ω*)^3/2^. The volume concentration of replicators in the daughters is homogeneous; therefore, the quantities of replicators are proportional to their volumes.

**Figure 4:**
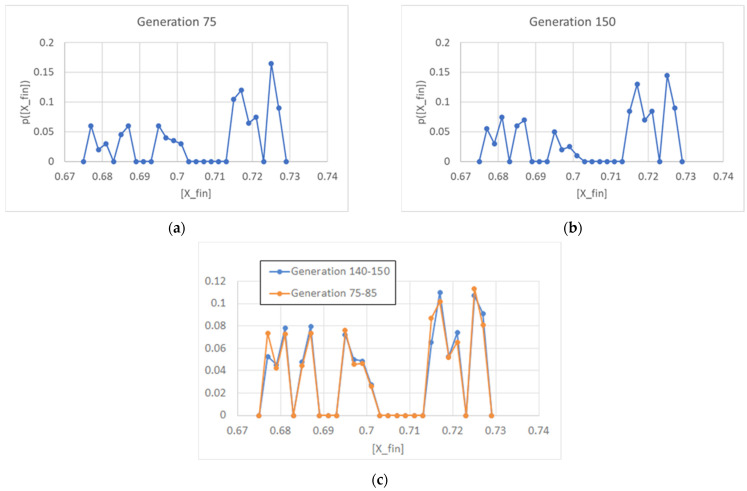
(**a**) Distribution of concentration of a linear replicator X at the 75th generation (15,000 duplications in a population of 200 individuals, asymmetrical division, *ω* = 0.4). (**b**) The same distribution at the 150th generation (30,000 duplications in a population of 200 individuals). (**c**) The same distribution computed on two samples of 10 generations, showing that the shape of the distributions is basically constant in time.

As in Section 3, let *ξ* be equal to *ω* for one daughter protocell and to (1 − *ω*) for the other one: then, in both cases, the relevant concentration, *X*/*V*, obeys the following equation:

(11) $$\frac{X_{initial}}{V_{initial}}=\frac{\xi^{3/2}X_{fin,previous}}{(\xi {\theta )}^{3/2}}=\frac{X_{fin,previous}}{V_{fin}}$$

which is identical to Equation (8). If the asymptotic *X_fin_* were the same in different generations, we would come to the same conclusions as in the previous section, but this is not the case. In the case of the linear self-replicator (described by the same Equation (2)), after several generations have passed, we continue to observe a distribution of different final values, contrary to what was observed in SRMs. This distribution (see Figure 4) seems to approach a constant non-obvious shape, where the final values tend to cluster in two groups, with a narrow gap in between.

**Figure 5:**
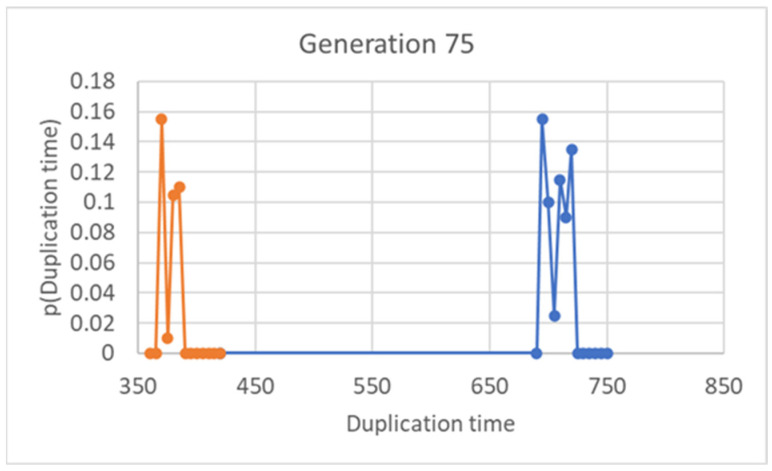
Duplication time at the 75th generation (15,000 duplications in a population of 200 individuals, asymmetrical division, *ω* = 0.4). We have highlighted in different colors two groups, corresponding to the duplication times of protocells born large (small duplication times, in orange color) and protocells born small (large duplication times, in blue color).

A bimodal distribution is observed also in duplication times (Figure 5), thus showing that the two groups are formed, as it should be expected, from protocells born either small or large (small ones take a longer time to reach the threshold for splitting).

**Figure 6:**
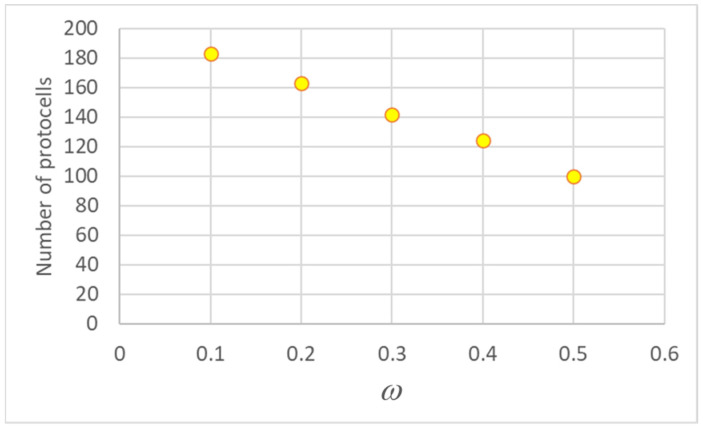
Number of “small” protocells as asymmetry varies: population of 200 protocells, 75th generation. The fraction of lipids inherited by the smaller descendant (i.e., *ω*) is shown on the x-axis.

The presence of two different duplication times involves an interesting consequence, leading in time to a higher number of protocells born small with respect to those that were born large. Indeed, in our simulations, whenever a “mother” protocell *M* reaches its critical size, it gives rise to two daughters, a large and a small one, which take the place of both the mother and another randomly chosen protocell (*R*): *M* and *R* disappear from the population, while a large and a small cell are added. If *M* is small and *R* is large, or vice versa, the fraction of large and small protocells remains the same. If *M* and *R* are both large then the total number of small cells increases, and the number of large ones decreases, while the opposite happens if *M* and *R* are both small. But large cells have shorter lifetimes, i.e., they die faster than small ones, so the chosen “mother” is more likely to be a large protocell. This leads to an unbalance between the two populations: in the case of a mature population, the number difference between the two types in the population, which is related to their lifetimes, increases as the degree of asymmetry (i.e., the value of |*ω* − 1/2|) increases (as shown in Figure 6).

**Figure 7:**
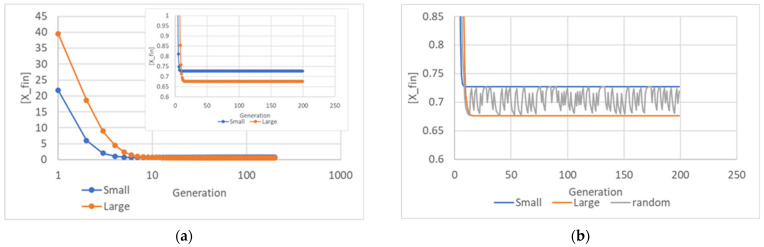
(**a**) Final concentration of *X* across generations, for the pure “large protocells only” and the pure “small protocells only” lineages (in the insert a magnification in linear scale). (**b**) Final concentration of *X* across generations of the two pure lineages, and of a lineage in which at each duplication only one randomly chosen protocell was followed. It can be noted that the concentration of protocells belonging to this “mixed” lineage, as the generations vary, varies between the extremes constituted by the concentrations of the “pure” lineage.

While we have so far examined static pictures taken at a given generation, or at most comparisons between pictures taken at different times, in the following Figure 7 we follow the value of the final concentration [*X_fin_*] through successive generations, starting from the very beginning. As generations pass, stories can become blurred. Remember that at each splitting there is one large and one small daughter. If we always follow the large daughter of the large daughter of the large daughter of the mother, we can see what happens to a “pure” lineage. As can be seen, the final concentrations become constant in this case. They also become constant in the case of a pure small lineage. If we show at each cell division the final concentration of a randomly chosen daughter, we see that the values oscillate (since the choice is random) but the oscillations do not take place only between the two pure values (Figure 7). This is again a consequence of the fact that in IRMs there is a distribution of final concentration values.

The above results (Figure 7) show that the total number of replicators in the two daughter protocells, born at different times, can differ from twice the value of the mother—a phenomenon that was not present in SRMs. Indeed, it is the average value of the replicator quantity that doubles, but the differences among generations give rise to oscillations.

Similar oscillations are also observed in the case of several linearly interacting replicators. Since the continuous equations are the same as those of SRMs, the ELRP and its eigenvectors play similar roles in IRMs. We show below the simulation of a two-replicator case, whose kinetic equations are as follows:

(12) $$\left\{\begin{array}{c} \frac{dC}{dt}=\alpha X\\ \frac{dX}{dt}=k_{11}X+k_{12}Y\\ \frac{dY}{dt}=k_{21}X+k_{22}Y \end{array}\right.$$

**Figure 8:**
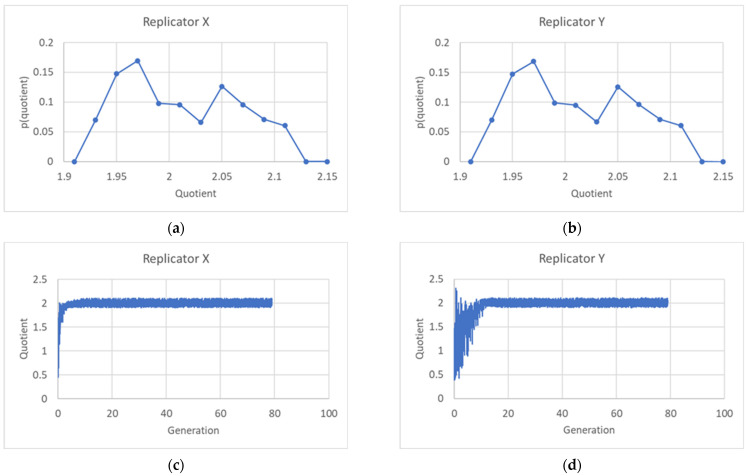
(**a**) Distribution of the ratio between the sum of the quantities of *X* of two “sister” protocells and the quantity of replicator *X* of the “mother” protocell at the 75th generation. (**b**) The same for the *Y* replicator. (**c**) Time trend of the ratio for the *X* replicator. (**d**) Time trend of the ratio for the *Y* replicator. In each protocell in (**c**,**d**), the ratio between the quantities of replicators is given by the corresponding ratios in the eigenvalue of the system.

The ratios *R* between the sum of final quantities of the two kinds of replicators, *X* and *Y*, in the daughter protocells, and the corresponding quantity in the mother may differ. We show in Figure 8 the distribution of these ratios, with 2 as the average value.

**Figure 9:**
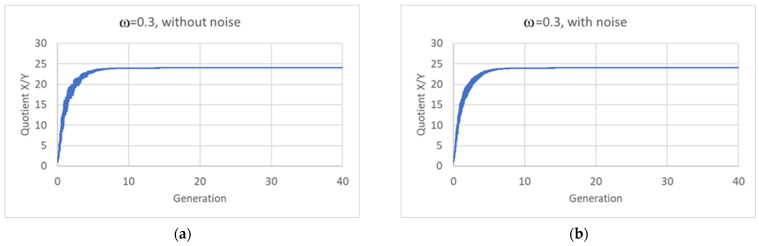
Ratio *X*/*Y* with the passing of generations, two linear replicators: (**a**) noise-free asymmetric division, ω = 0.3; (**b**) asymmetric division with noise (*ω* = 0.3 ± 0.1, with uniform distribution). In both plots, the quotient reaches exactly the ratio present in the eigenvector of the system (*k*_11_ = 5 × 10^−3^; *k*_12_ = 2 × 10^−6^; *k*_21_ = 2 × 10^−4^; *k*_22_ = 2 × 10^−4^; ratio= 24.0002).

The fact that the average value of the ratio *R* between the quantities is not always 2 does not make sustained growth impossible, since the constancy of its average value suffices to guarantee the maintenance of the protogenetic material. The identity of a protocell with several different replicators is rather associated with its chemical composition, which is directly related to the ratio between the quantities of the two replicators (or to the sets of ratios in the case of more than two replicators) rather than to the ratio *R* between total quantities in daughters and mother. And it is impressive to see how, at the time of division in a (mature) population, notwithstanding the oscillations of the various variables, this ratio is exactly the same, up to an impressive number of significant digits, for every protocell (Figure 9a). The relative composition of the protogenetic material remains the same, so homogeneous growth actually takes place in the model.

Figure 9b looks identical to Figure 9a: this surprising property will be commented on in the final Section 5.

## 5. Discussion and Indications for Further Work

In this paper, we generalize the notion of synchronization, which had been previously applied to the study of symmetrical division, to that of “homogeneous growth”, which assures the sustainable growth of a population of protocells undergoing asymmetrical division. The main conclusion of our studies is that, in those models that have been analyzed, such homogeneous growth can be observed under a broad set of conditions: in particular, we have found that homogenous growth under asymmetrical division takes place in all those cases where the kinetic equations for the replicators and for their coupling to the growth of the lipid container would lead to synchronization under symmetrical division. 

Here, we have adapted to asymmetrical division the same modeling framework that had been used in our previous studies. This is indeed a fairly abstract view of a protocell, which is essentially described by a set of coupled kinetic equations for the replicators and for the lipid container. We found that several detailed models of protocells fit this framework, using different kinetic equations: therefore, we examined the model behaviors under a wide set of such equations, and we discovered that synchronization is surprisingly common. It is crystal clear that there are several major problems concerning protocells that cannot be dealt with at such a high abstraction level, including (but not limited to) the chemical nature of replicators, the details of their dynamics, their coupling to lipid synthesis, and the mechanism of cell division. These and other problems lie beyond the scope of these studies, as discussed in more detail in our previous work (see in particular [21,31,32]. However, in spite of these limitations, we are also convinced that high-level, abstract models like those described here, which show that some important properties are quite widespread, provide interesting and useful knowledge, complementary to that of more specific models and applicable to different specific hypotheses. In particular, in studies on the origin of life, different alternative specific hypotheses have been and are being proposed (concerning the architecture of protocells, the chemical nature of replicators, their coupling to lipid synthesis, the mechanism of cell fission, etc.); therefore, the availability of results that hold under a broad range of conditions provides support to scenarios that include protocells: some properties, like homogeneous growth, can often be assumed to have been proven a priori.

The achievement of homogeneous growth is particularly clear in the case of Surface Reaction Models. We stress that this result is not trivial, since the lifetime (i.e., the time interval from their birth to their splitting) of those protocells that are born small is larger than that of their larger “sisters”. In the case of linear replicators, after a transient has died out, the pairs of newborn protocells tends to be equal in successive generations. This is a consequence of the fact that the concentration of replicators at division times tend to become equal—exactly as it happens in the case of symmetrical division.

In the case of asymmetrical division of IRMs, these equalities no longer hold. As we have seen, the initial concentration of replicators in pairs of newborn protocells can be different from that of their parent, even after many generations, so that quantities of replicators are not identical in successive generations. However, we have argued that the chemical identity of a protocell is not related to the volume concentration of the replicators, but rather to their ratios, and we have shown that this ratio tends to be remarkably constant in every protocell of a population.

An important question concerns the robustness of these results with respect to random fluctuations in the sizes of the daughter protocells. Fission and budding processes can be affected by several uncontrolled variables, so it may be unrealistic to assume that division always takes place at a given critical size. This point needs to be addressed in a wider future study, but we have analyzed the case of two linear replicators (Equation (12)), supposing that splitting gives rise to a large and to a small protocell, with *ω* chosen at random, with uniform probability, in the interval [0.2,0.4]. The results are shown in Figure 9b, which looks the same as its counterpart with a fixed value *ω* = 0.30. Homogeneous growth is maintained also when size fluctuations are taken into account.

These results have been achieved in the case of linear kinetic equations, and they still need to be analyzed under more general assumptions. We have also successfully explored some nonlinear cases; however, the possibility of homogeneous growth needs to be verified in a broader set of cases. In any case, this paper demonstrates its validity under a wide set of interesting hypotheses.

There are of course several improvements that are worth exploring, while still keeping the present high abstraction level, and we plan to address them in future works. A particularly important modification concerns the removal of the hypothesis of instantaneously buffered precursors, in favor of that of a finite transmembrane diffusion rate. This had already been done in our previous work with symmetrical division, where it seems that synchronization is easier to achieve when such approximation is removed: we have studied models with finite diffusion rates [21,40], showing that they achieve synchronization even in cases that did not do so with an infinite diffusion rate (like e.g., quadratic equations for self-replicators). In future works, we will analyze the case of asymmetrical division in the same way.

We are also interested in considering the possibility of placing a protocell population in a flow reactor in order to prevent its unbounded growth without imposing a sharp fixed size. Other improvements that we plan to consider include possible osmotic effects (discussed in [23,24,25]), the introduction of an age-dependent probability of removal of existing protocells and the possibility that some replicators are found in the membrane and some in the internal water phase. We also plan to examine the possibility of using a more articulated model of the fission process; however, it is not presently clear that this problem can be addressed without restricting the treatment to quite detailed protocell models (thus abandoning in some way the present level of abstraction).

A final word of caution concerns the fact that the duplication process described here is quite different from that of present-day cells. In this latter case, not only are there effective controls that guarantee that the genetic material has been duplicated before fission, but the whole process of DNA duplication is made in such a way as to generate, with high probability, macromolecules that are identical to those of the parent. It seems unlikely that such a process emerged all at once. The mechanisms described in this paper, where molecules proliferate under kinetic equations, are plausible for protocells at the dawn of life, but they must have been overtaken at some time to generate those that are at work nowadays. Understanding this takeover (possibly a new major transition in evolution) is an open, fascinating problem.

## Data Availability

Some of the materials presented can be accessed at the link: https://drive.google.com/file/d/1wqvdvdpdlBgQNcLRap3LJoWSaQ-96Z3y/view?usp=sharing.

## Appendix A

In Table A1 you can find the list of the main symbols used in the text, with a brief explanation of their meaning.

**Table A1:** List of symbols used in the text.

Symbol	Description
C	Quantity of lipids within the membrane of the protocell
$\vec{X}$	Quantities of the q types of replicators *X*_1_ *…X_q_*
V	Total volume of the protocell
S	Surface area of the protocell
θ	Quantity of lipids corresponding to the fission of the protocell
δ	Membrane width
ρ	Lipid density
$\alpha ,\;\vec{\alpha }$	Coupling coefficients between internal materials and lipid growth
η, M, K	Catalysis coefficients of the internal material
${\Delta T}_{k}$	Duplication time at the kth generation
ω, (1 − ω), *ξ*	In asymmetric division, the fraction of lipids inherited by each of the daughter protocells (*ξ* stand for “either fraction”)

## References

[1] Alberts B., Johnson A., Lewis J., Morgan D., Raff M., Roberts K., Walter P. (2014). Molecular Biology of the Cell.

[2] Luisi P.L. (2007). The Emergence of Life: From Chemical Origins to Synthetic Biology.

[3] Rasmussen S., Bedau M.A., Chen L., Deamer D., Krakauer D.C., Packard N.H., Stadler P.F. (2008). Protocells.

[4] Rasmussen S., Constantinescu A., Svaneborg C. (2016). Generating minimal living systems from non-living materials and increasing their evolutionary abilities. Philos. Trans. R. Soc. Lond. B.

[5] Gánti T. (2003). Chemoton Theory: Theory of Living Systems.

[6] Kaneko K. (2006). Life: An Introduction to Complex Systems Biology.

[7] Mavelli F., Ruiz-Mirazo K. (2007). Stochastic simulations of minimal self-reproducing cellular systems. Philos. Trans. R. Soc. Lond. B Biol. Sci..

[8] Israelachvili J.N., Mitchell D.J., Ninham B.W. (1976). Theory of self-assembly of hydrocarbon amphiphiles into micelles and bilayers. J. Chem. Soc. Faraday Trans..

[9] Israelachvili J.N., Mitchell D.J., Ninham B.W. (1977). Theory of self-assembly of lipid bilayers and vesicles. Biochim. Biophys. Acta.

[10] Sakuma Y., Imai M., Stano P., Mavelli F. (2015). From vesicles to protocells: The roles of amphiphilic molecules. Life.

[11] Szostak J.W., Bartel D.P., Luisi P.L. (2001). Synthesizing life. Nature.

[12] Walde P., Umakoshi H., Stano P., Mavelli F. (2014). Emergent properties arising from the assembly of amphiphiles. Artificial vesicle membranes as reaction promoters and regulators. Chem. Commun..

[13] Hanczyc M.M., Szostak J.W. (2004). Replicating vesicles as models of primitive cell growth and division. Curr. Opin. Chem. Biol..

[14] Terasawa H., Nishimura K., Suzuki H., Matsuura T., Yomo T. (2012). Coupling of the fusion and budding of giant phospholipid vesicles containing macromolecules. Proc. Natl. Acad. Sci. USA.

[15] Svetina S. (2009). Vesicle budding and the origin of cellular life. ChemPhysChem.

[16] Svetina S. (2012). Cellular life could have emerged from properties of vesicles. Orig. Life Evol. Biosph..

[17] Miele Y., Holló G., Lagzi I., Rossi F. (2022). Shape Deformation, Budding and Division of Giant Vesicles and Artificial Cells: A Review. Life.

[18] Filisetti A., Serra R., Carletti T., Poli I., Villani M. (2008). Synchronization phenomena in protocell models. Biophys. Rev. Lett..

[19] Serra R., Filisetti A., Villani M., Graudenzi A., Damiani C., Panini T. (2014). A stochastic model of catalytic reaction networks in protocells. Nat. Comput..

[20] Villani M., Filisetti A., Graudenzi A., Damiani C., Carletti T., Serra R. (2014). Growth and division in a dynamic protocell model. Life.

[21] Serra R., Villani M. (2017). Modelling Protocells.

[22] Moran P.A.P. (1958). Random processes in genetics. Mathematical Proceedings of the Cambridge Philosophical Society.

[23] Mavelli F., Ruiz-Mirazo K. (2013). Theoretical conditions for the stationary reproduction of model protocells. Integr. Biol..

[24] Bigan E., Steyaert J.M., Douady S. (2015). Minimal conditions for protocell stationary growth. Artif. Life.

[25] Higgs P.G. (2021). When Is a Reaction Network a Metabolism? Criteria for Simple Metabolisms That Support Growth and Division of Protocells. Life.

[26] Munteanu A., Attolini C.S.O., Rasmussen S., Ziock H., Solé R.V. (2007). Generic Darwinian selection in catalytic protocell assemblies. Philos. Trans. R. Soc. Lond. B Biol. Sci..

[27] Rasmussen S., Chen L., Nilsson M., Abe S. (2003). Bridging nonliving and living matter. Artif. Life.

[28] Rasmussen S., Chen L., Deamer D., Krakauer D.C., Packard N.H., Stadler P.F., Bedau M.A. (2004). Transitions from nonliving to living matter. Science.

[29] Rasmussen S., Chen L., Stadler B.M.R., Stadler P.F. (2004). Photo-organism kinetics: Evolutionary dynamics of lipid aggregates with genes and metabolism. Orig. Life Evol. Biosph..

[30] McCaskill J.S., Packard N.H., Rasmussen S., Bedau M.A. (2007). Evolutionary self-organization in complex fluids. Philos. Trans. R. Soc. Lond. B Biol. Sci..

[31] Serra R., Carletti T., Poli I. (2007). Synchronization phenomena in surface-reaction models of protocells. Artif. Life.

[32] Carletti T., Serra R., Poli I., Villani M., Filisetti A. (2008). Sufficient conditions for emergent synchronization in protocell models. J. Theor. Biol..

[33] Villani M., Filisetti A., Nadini M., Serra R., Rossi F., Mavelli F., Stano P., Caivano D. (2016). On the Dynamics of Autocatalytic Cycles in Protocell Models. Advances in Artificial Life, Evolutionary Computation and Systems Chemistry, Proceedings of the WIVACE 2015, Bari, Italy, 23–24 September 2015.

[34] Filisetti A., Serra R., Carletti T., Villani M., Poli I. (2010). Non-linear protocell models: Synchronization and chaos. Eur. Phys. J. B..

[35] Segré D., Lancet D., Kedem O., Pilpel Y. (1998). Graded Autocatalysis Replication Domain (GARD): Kinetic analysis of self-replication in mutually catalytic sets. Orig. Life Evol. Biosph..

[36] Segré D., Ben-Eli D., Deamer D.W., Lancet D. (2001). The lipid world. Orig. Life Evol. Biosph..

[37] Hordijk W., Steel M. (2004). Detecting autocatalytic, self-sustaining sets in chemical reaction systems. J. Theor. Biol..

[38] Hordijk W., Hein J., Steel M. (2010). Autocatalytic sets and the origin of life. Entropy.

[39] Musa M., Villani M., Serra R., Pelillo M., Poli I., Roli A., Serra R., Slanzi D., Villani M. (2018). Simulating Populations of Protocells with Uneven Division. Proceedings of the Artificial Life and Evolutionary Computation, WIVACE 2017.

[40] Serra R., Villani M. (2019). Sustainable Growth and Synchronization in Protocell Models. Life.

